# Supplementary material for: Eliminating bias: enhancing children’s book recommendation using a hybrid model of graph convolutional networks and neural matrix factorization
Source: PeerJ Comput Sci. 2024 Feb 29;10:e1858. doi: 10.7717/peerj-cs.1858 (PMC10909171; doi:10.7717/peerj-cs.1858)
Supplement: Supplemental Information 1 [file peerj-cs-10-1858-s001.pdf]

In [1]:
```
import numpy as np
import pandas as pd
import matplotlib.pyplot as plt
import seaborn as sns
```

In [2]:
```
data1 = pd.read_csv('data1.csv')
data2 = pd.read_csv('data2.csv')
data3 = pd.read_csv('Review.csv')
```

In [3]:
```
fig, axs = plt.subplots(1, 2, figsize=(12, 6))
hb1 = axs[0].scatter(data3['average_score'], data3['Audience Score'], c=t
axs[0].set_xlabel('Top Critics Score')
axs[0].set_ylabel('Audience Score')
axs[0].set_title('Audience Score vs Top Critics Score')
cb1 = plt.colorbar(hb1, ax=axs[0])
cb1.set_label('Density')

axs[0].plot([0, 1], [0, 1], color='black', linestyle='--')
hb2 = axs[1].scatter(data3['average_score'], data3['Critics Score'], c=to
axs[1].set_xlabel('Top Critics Score')
axs[1].set_ylabel('Critics Score')
axs[1].set_title('Critics Score vs Top Critics Score')
cb2 = plt.colorbar(hb2, ax=axs[1])
cb2.set_label('Density')

axs[1].plot([0, 1], [0, 1], color='black', linestyle='--')
plt.tight_layout()
plt.savefig("1.pdf")
plt.show()
```

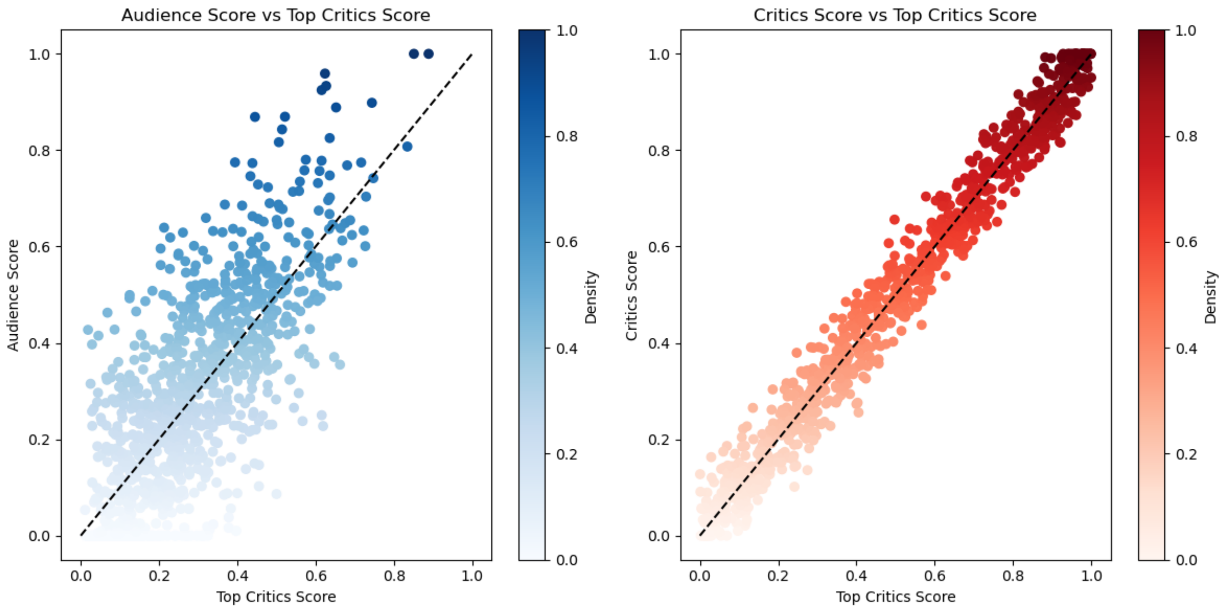

In [4]:
```
df1 = pd.DataFrame(data1)
df2 = pd.DataFrame(data2)

plt.figure(figsize=(8, 6))
sns.set(style='whitegrid', font_scale=1.2)
sns.lineplot(x='Books Count', y='Rating Count', data=df1, label='Dataset1
sns.lineplot(x='Books Count', y='Rating Count', data=df2, label='Dataset2
plt.xlabel('Books Count')
plt.ylabel('Rating Count')
plt.legend(loc='upper right', fontsize=12)
plt.title('Children Books Count vs Rating Count Comparison', fontsize=14)
plt.savefig("2.pdf")
plt.show()
```

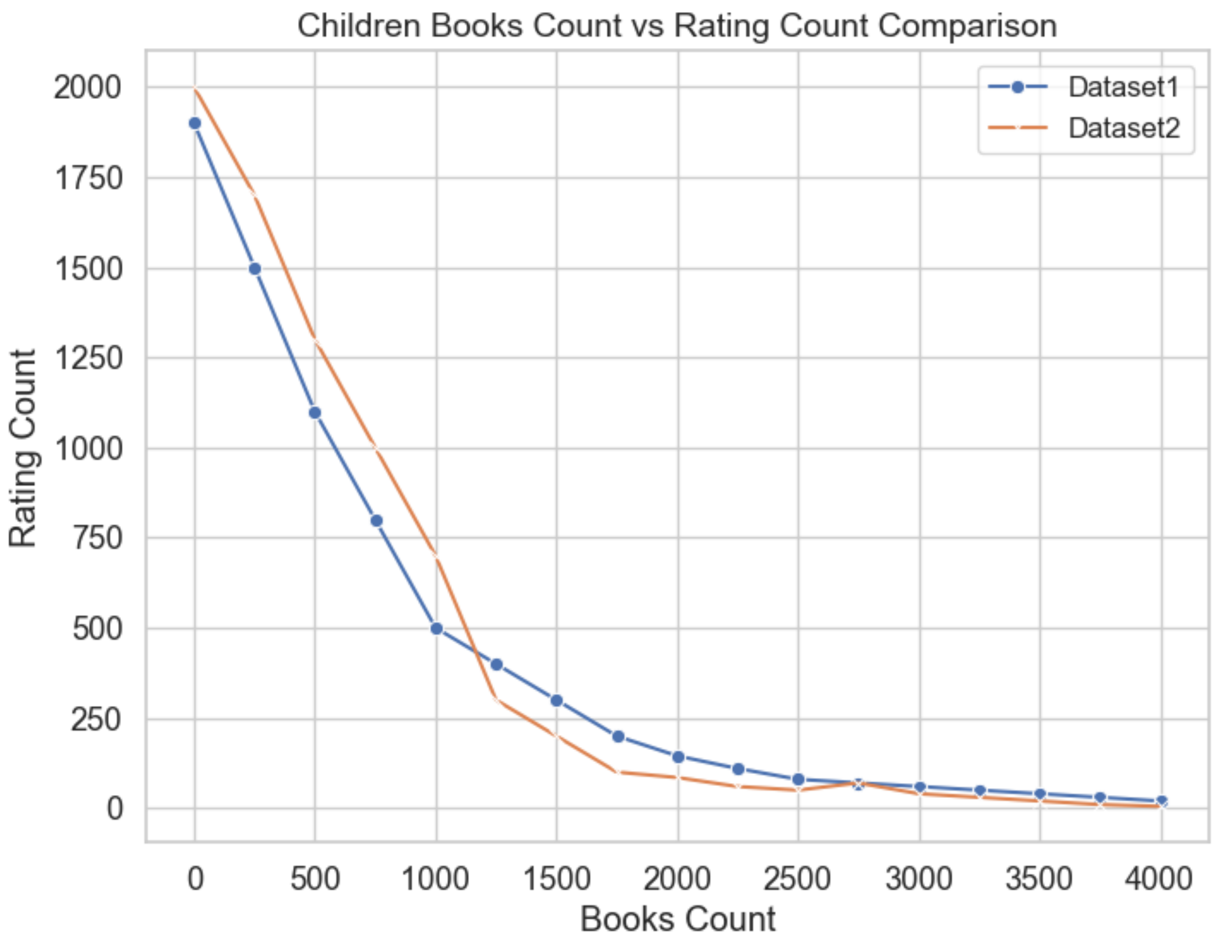

In [5]:
```
import torch
import numpy as np
import torch.nn as nn
from torch import optim
import matplotlib.pyplot as plt
from scipy.sparse import csr_matrix
from torch.nn import functional as F
from torch_geometric.data import Data
from torch_geometric.nn import GCNConv
from scipy.sparse.csgraph import bipartite
from torchvision import datasets, transforms
from torch.utils.data import DataLoader, Dataset
from sklearn.metrics import mean_absolute_error, mean_squared_error
```

In [6]:

```
class Mydataset1(Dataset):
    def __init__(self,mode,transform = None):
        self.X,self.Y= self.load_csv('data1.csv')
        if mode == 'train':  # 75%
            self.data = self.X[:int(0.75 * self.total_len)]
            self.labels = self.Y[:int(0.75*self.total_len)]
        elif mode == 'valid':  # 25%
            self.data = self.X[int(0.75*self.total_len):]
            self.labels = self.Y[int(0.75*self.total_len):]

    def load_csv(self,root):

        data= pd.read_csv(root)
        data=data.replace(np.NaN,0)
        f=data.iloc[:,1:]
        sequence,X,Y = 1,[],[]
        for i in range(df.shape[0] - sequence):
            X.append(np.array(df.iloc[i:(i+sequence),].values,dtype=np.fl
            Y.append(np.array(df.iloc[i,0],dtype=np.float32))
        X = np.array(X)
        Y = np.array(Y)
        Y = np.expand_dims(Y, 1)
        return X,Y

    def __len__(self):
        return len(self.data)

    def __getitem__(self, index):
        x1 = self.data[index]
        y1=  self.labels[index]
        return torch.tensor(x1),torch.tensor(y1)

train_dataset1= Mydataset1('train')
test_dataset1 = Mydataset1('valid')
```

In [7]:
```
class Mydataset2(Dataset):
    def __init__(self,mode,transform = None):
        self.X,self.Y= self.load_csv('data2.csv')
        if mode == 'train':  # 75%
            self.data = self.X[:int(0.75 * self.total_len)]
            self.labels = self.Y[:int(0.75*self.total_len)]
        elif mode == 'valid':  # 25%
            self.data = self.X[int(0.75*self.total_len):]
            self.labels = self.Y[int(0.75*self.total_len):]

    def load_csv(self,root):
        data= pd.read_csv(root)
        data=data.replace(np.NaN,0)
        f=data.iloc[:,1:]
        sequence,X,Y = 1,[],[]
        for i in range(df.shape[0] - sequence):
            X.append(np.array(df.iloc[i:(i+sequence),].values,dtype=np.fl
            Y.append(np.array(df.iloc[i,0],dtype=np.float32))
        X = np.array(X)
        Y = np.array(Y)
        Y = np.expand_dims(Y, 1)
        return X,Y

    def __len__(self):
        return len(self.data)

    def __getitem__(self, index):
        x1 = self.data[index]
        y1=  self.labels[index]
        return torch.tensor(x1),torch.tensor(y1)

train_dataset2= Mydataset2('train')
test_dataset2 = Mydataset2('valid')
```

In [8]:
```
train_data1 = DataLoader(train_dataset1, batch_size=16, shuffle=True)
train_data2 = DataLoader(train_dataset2, batch_size=16, shuffle=True)
test_data1 = DataLoader(test_dataset1, batch_size=16, shuffle=True)
test_data2 = DataLoader(test_dataset2, batch_size=16, shuffle=True)
```

Our method

In [9]:
```
def nmf(R, Z, _lambda, T, learning_rate, decay_rate):
    num_users, num_items = R.shape
    num_features = Z.shape[1]
    P = np.random.rand(num_users, num_features)
    Q = np.random.rand(num_items, num_features)
    for t in range(T):
        for i in range(num_users):
            for j in range(num_items):
                if R[i, j] > 0:
                    e_ij = R[i, j] - np.dot(P[i, :], Q[j, :].T)
                    P[i, :] = P[i, :] + 2 * learning_rate * (e_ij * Q[j,
                    Q[j, :] = Q[j, :] + 2 * learning_rate * (e_ij * P[i,
        learning_rate = learning_rate * (1 - decay_rate)
    return P, Q

class Our_Module(nn.Module):
    def __init__(self, A, X, num_classes):
        super(Our_Module, self).__init__()
        self.A = torch.from_numpy(A).float()
        self.X = torch.from_numpy(X).float()
        self.num_classes = num_classes
        self.conv1 = GCNConv(self.X.shape[1], 16)
        self.conv2 = GCNConv(16, num_classes)


    def forward(self, edge_index):
        x = self.X
        x = self.conv1(x, edge_index)
        x = F.relu(x)
        x = F.dropout(x, training=self.training)
        x = self.conv2(x, edge_index)
        return F.log_softmax(x, dim=1)

A = np.array([[0, 1, 0], [1, 0, 1], [0, 1, 0]])
X = np.array([[0, 1, 0], [1, 0, 1], [0, 1, 0]])
num_classes = 1
R = np.array([[5, 3, 0, 1], [4, 0, 0, 1], [1, 1, 0, 5], [1, 0, 0, 4], [0,
Z = np.array([[0.1, 0.2], [0.2, 0.1], [0.3, 0.4], [0.4, 0.3], [0.5, 0.6]]
_lambda = 0.1
T = 100
learning_rate = 0.01
decay_rate = 0.01
P, Q = nmf(R, Z, _lambda, T, learning_rate, decay_rate)
num_features = P.shape[1] + Q.shape[1]
num_features = 64
num_classes = 1
Our_Module = Our_Module( A, X, num_classes)
optimizer = optim.Adam(Our_Module.parameters(), lr=0.01)
A = np.zeros((R.shape[0], R.shape[1]))
for i in range(R.shape[0]):
    for j in range(R.shape[1]):
        if R[i, j] > 0:
            A[i, j] = 1
```

In [10]:

```
def Our_Module_train1():
    Our_model.train()
    for epoch in range(200):
        for batch_idx, (data, target) in enumerate(train_data1):
            optimizer.zero_grad()
            input_data = torch.tensor(np.concatenate([P, Q], axis=1), dty
            target_data = torch.tensor(R, dtype=torch.float)
            out = Our_Module(torch.tensor(A, dtype=torch.float), data)
            loss = F.mse_loss(out[data.train_mask].flatten(), data.y[data
            loss.backward()
            optimizer.step()

def Our_Module_test1():
    Our_model.eval()#Model testing
    Pred,Real,Bias=[],[],[]
    with torch.no_grad():
        for data, target in test_data1:
            output = Our_Model(data)
            test_loss += criterion(output, target).item()
            pred = output.argmax(1, keepdim=True)
            correct += pred.eq(target.view_as(pred)).sum().item()# add th
            real_values = data.y[data.test_mask]
            Mean = target.mean()-pred.mean()
            bias = (preds[data.test_mask] - real_values).mean()
            Pred.append(pred)
            Real.append(real_values)
            Bias.append(bias)
        mse = mean_squared_error(real_values.cpu().detach().numpy(), pred
        mae = mean_absolute_error(real_values.cpu().detach().numpy(), pre
        accuracy = 100. * correct / len(test_data1.dataset)
    test_loss /= len(test_loader.dataset)
    return mse ,mae,Bias,accuracy,test_loss,Pred,Real,Mean

round=20
Our_Module_Mse1=[]
Our_Module_Mae1=[]
Our_Module_Bias1=[]
Our_Module_Accuracy1=[]
Our_Module_Test_loss1=[]
Our_Module_Pred1=[]
Our_Module_Real1=[]
Our_Module_Mean1=[]
for i in range(round):
    Our_Module_train1()
    Our_Module_mse1 ,Our_Module_mae1,Our_Module_bias1,Our_Module_accuracy
    Our_Module_Mse1.append( Our_Module_mse1 )
    Our_Module_Mae1.append(Our_Module_mae1)
    Our_Module_Bias1.append(Our_Module_bias1)
    Our_Module_Accuracy1.append(Our_Module_accuracy1)
    Our_Module_Test_loss1.append(Our_Module_test_loss1)
    Our_Module_Pred1.append(Our_Module_pred1)
    Our_Module_Real1.append(Our_Module_real1)
    Our_Module_Mean1.append(Our_Module_mean1)
    print('\nTest {} round set: Average loss for {:.3f} , Accuracy: {:.0f
        i+1,Our_Module_test_loss1[i], Our_Module_accuracy1[i]))
```

```
Test 1 round set: Average loss for 0.381 , Accuracy: 62%

Test 2 round set: Average loss for 0.272 , Accuracy: 73%

Test 3 round set: Average loss for 0.225 , Accuracy: 78%

Test 4 round set: Average loss for 0.185 , Accuracy: 82%

Test 5 round set: Average loss for 0.144 , Accuracy: 86%

Test 6 round set: Average loss for 0.105 , Accuracy: 90%

Test 7 round set: Average loss for 0.082 , Accuracy: 92%

Test 8 round set: Average loss for 0.079 , Accuracy: 93%

Test 9 round set: Average loss for 0.063 , Accuracy: 94%

Test 10 round set: Average loss for 0.056 , Accuracy: 95%

Test 11 round set: Average loss for 0.044 , Accuracy: 96%

Test 12 round set: Average loss for 0.036 , Accuracy: 97%

Test 13 round set: Average loss for 0.027 , Accuracy: 98%

Test 14 round set: Average loss for 0.024 , Accuracy: 98%

Test 15 round set: Average loss for 0.022 , Accuracy: 98%

Test 16 round set: Average loss for 0.015 , Accuracy: 98%

Test 17 round set: Average loss for 0.015 , Accuracy: 98%

Test 18 round set: Average loss for 0.015 , Accuracy: 99%

Test 19 round set: Average loss for 0.015 , Accuracy: 99%
```

```
Test 20 round set: Average loss for 0.014 , Accuracy: 99%
```

In [10]:

```
def Our_Module_train2():
    # Training to Winnnnnnnnnnnnnnnnnnnnnnnnnn
    Our_model.train()
    for epoch in range(200):
        for batch_idx, (data, target) in enumerate(train_data2):
            optimizer.zero_grad()
            input_data = torch.tensor(np.concatenate([P, Q], axis=1), dty
            target_data = torch.tensor(R, dtype=torch.float)
            out = Our_Module(torch.tensor(A, dtype=torch.float), data)
            loss = F.mse_loss(out[data.train_mask].flatten(), data.y[data
            loss.backward()
            optimizer.step()
def Our_Module_test2():
    Our_Module.eval()#Testing again
    Pred,Real,Bias=[],[],[]
    with torch.no_grad():
        for data, target in test_data2:
            output = Our_Module(data)
            test_loss += criterion(output, target).item()
            pred = output.argmax(1, keepdim=True)
            correct += pred.eq(target.view_as(pred)).sum().item()# one by
            real_values = data.y[data.test_mask]
            Mean = target.mean()-pred.mean()
            bias = (preds[data.test_mask] - real_values).mean()
            Pred.append(pred)
            Real.append(real_values)
            Bias.append(bias)
        mse = mean_squared_error(real_values.cpu().detach().numpy(), pred
        mae = mean_absolute_error(real_values.cpu().detach().numpy(), pre
        accuracy = 100. * correct / len(test_data1.dataset)
    test_loss /= len(test_loader.dataset)
    return mse ,mae,Bias,accuracy,test_loss,Pred,Real,Mean

round=20
Our_Module_Mse2=[]
Our_Module_Mae2=[]
Our_Module_Bias2=[]
Our_Module_Accuracy2=[]
Our_Module_Test_loss2=[]
Our_Module_Pred2=[]
Our_Module_Real2=[]
Our_Module_Mean2=[]
for i in range(round):
    Our_Module_train2()
    Our_Module_mse2 ,Our_Module_mae2,Our_Module_bias2,Our_Module_accuracy
    Our_Module_Mse2.append( Our_Module_mse2 )
    Our_Module_Mae2.append(Our_Module_mae2)
    Our_Module_Bias2.append(Our_Module_bias2)
    Our_Module_Accuracy2.append(Our_Module_accuracy2)
    Our_Module_Test_loss2.append(Our_Module_test_loss2)
    Our_Module_Pred2.append(Our_Module_pred2)
    Our_Module_Real2.append(Our_Module_real2)
    Our_Module_Mean2.append(Our_Module_mean2)
    print('\nTest {} round set: Average loss for {:.3f} , Accuracy: {:.0f
        i+1,Our_Module_Test_loss2[i], Our_Module_accuracy2[i]))
```

```
Test 1 round set: Average loss for 0.481 , Accuracy: 52%

Test 2 round set: Average loss for 0.372 , Accuracy: 63%

Test 3 round set: Average loss for 0.264 , Accuracy: 74%

Test 4 round set: Average loss for 0.183 , Accuracy: 82%

Test 5 round set: Average loss for 0.148 , Accuracy: 86%

Test 6 round set: Average loss for 0.145 , Accuracy: 90%

Test 7 round set: Average loss for 0.088 , Accuracy: 92%

Test 8 round set: Average loss for 0.074 , Accuracy: 93%

Test 9 round set: Average loss for 0.068 , Accuracy: 94%

Test 10 round set: Average loss for 0.055 , Accuracy: 95%

Test 11 round set: Average loss for 0.049 , Accuracy: 96%

Test 12 round set: Average loss for 0.036 , Accuracy: 97%

Test 13 round set: Average loss for 0.033 , Accuracy: 97%

Test 14 round set: Average loss for 0.025 , Accuracy: 98%

Test 15 round set: Average loss for 0.024 , Accuracy: 98%

Test 16 round set: Average loss for 0.023 , Accuracy: 98%

Test 17 round set: Average loss for 0.022 , Accuracy: 98%

Test 18 round set: Average loss for 0.021 , Accuracy: 98%

Test 19 round set: Average loss for 0.017 , Accuracy: 98%
```

```
Test 20 round set: Average loss for 0.015 , Accuracy: 98%
```

Our method without NMF

In [11]:
```
class NO_NMF(nn.Module):
    def __init__(self, A, X, num_classes):
        super(NO_NMF, self).__init__()
        self.A = torch.from_numpy(A).float()
        self.X = torch.from_numpy(X).float()
        self.num_classes = num_classes
        self.conv1 = GCNConv(self.X.shape[1], 16)
        self.conv2 = GCNConv(16, num_classes)

    def forward(self, edge_index):
        x = self.X
        x = self.conv1(x, edge_index)
        x = F.relu(x)
        x = F.dropout(x, training=self.training)
        x = self.conv2(x, edge_index)
        return F.log_softmax(x, dim=1)

A = np.array([[0, 1, 0], [1, 0, 1], [0, 1, 0]])
X = np.array([[0, 1, 0], [1, 0, 1], [0, 1, 0]])
num_classes = 1

NO_NMF_model = NO_NMF(A, X, num_classes)
optimizer = optim.SGD(NO_NMF_model.parameters(), lr=0.01)
```

In [12]:
```
def NO_NMF_train1():
    # 1
    NO_NMF_model.train()
    for epoch in range(200):
        for batch_idx, (data, target) in enumerate(train_data1):
            optimizer.zero_grad()
            out = NO_NMF_model(data,A_tensor, X_tensor)
            loss = F.mse_loss(out[data.train_mask].flatten(), data.y[data
            loss.backward()
            optimizer.step()

def NO_NMF_test1():
    NO_NMF_model.eval()#2
    Pred,Real.Bias=[],[],[]
    with torch.no_grad():
        for data, target in test_data1:
            output = NO_NMF_model(data,A_tensor, X_tensor)
            test_loss += criterion(output, target).item()
            pred = output.argmax(1, keepdim=True)
            correct += pred.eq(target.view_as(pred)).sum().item()#3
            real_values = data.y[data.test_mask]
            Mean = target.mean()-pred.mean()
            bias = (preds[data.test_mask] - real_values).mean()
            Pred.append(pred)
            Real.append(real_values)
            Bias.append(bias)
        mse = mean_squared_error(real_values.cpu().detach().numpy(), pred
        mae = mean_absolute_error(real_values.cpu().detach().numpy(), pre
        bias = (preds[data.test_mask] - real_values).mean()
        accuracy = 100. * correct / len(test_data1.dataset)
    test_loss /= len(test_loader.dataset)
    return mse,mae,Bias,accuracy,test_loss,Pred,Real,Mean

round=20
NO_NMF_Mse1=[]
NO_NMF_Mae1=[]
NO_NMF_Bias1=[]
NO_NMF_Accuracy1=[]
NO_NMF_Test_loss1=[]
NO_NMF_Pred1=[]
NO_NMF_Real1=[]
NO_NMF_Mean1=[]
for i in range(round):
    NO_NMF_train1()
    NO_NMF_mse1,NO_NMF_mae1,NO_NMF_bias1,NO_NMF_accuracy1,NO_NMF_test_los
    NO_NMF_Mse1.appendNO_NMF_mse1)
    NO_NMF_Mae1.append(NO_NMF_mae1)
    NO_NMF_Bias1.append(NO_NMF_bias1)
    NO_NMF_Accuracy1.append(NO_NMF_accuracy1)
    NO_NMF_Test_loss1.append(NO_NMF_test_loss1)
    NO_NMF_Pred1.append(NO_NMF_pred1)
    NO_NMF_Real1.append(NO_NMF_real1)
    NO_NMF_Mean1.append(NO_NMF_mean1)
    print('\nTest {} round set: Average loss for {:.3f} , Accuracy: {:.0f
        i+1,NO_NMF_Test_loss1[i], NO_NMF_accuracy1[i]))
```

```
Test 1 round set: Average loss for 0.383 , Accuracy: 62%

Test 2 round set: Average loss for 0.284 , Accuracy: 72%

Test 3 round set: Average loss for 0.255 , Accuracy: 75%

Test 4 round set: Average loss for 0.229 , Accuracy: 78%

Test 5 round set: Average loss for 0.184 , Accuracy: 82%

Test 6 round set: Average loss for 0.162 , Accuracy: 84%

Test 7 round set: Average loss for 0.127 , Accuracy: 88%

Test 8 round set: Average loss for 0.110 , Accuracy: 90%

Test 9 round set: Average loss for 0.093 , Accuracy: 91%

Test 10 round set: Average loss for 0.084 , Accuracy: 92%

Test 11 round set: Average loss for 0.074 , Accuracy: 93%

Test 12 round set: Average loss for 0.066 , Accuracy: 94%

Test 13 round set: Average loss for 0.059 , Accuracy: 95%

Test 14 round set: Average loss for 0.052 , Accuracy: 95%

Test 15 round set: Average loss for 0.045 , Accuracy: 96%

Test 16 round set: Average loss for 0.043 , Accuracy: 96%

Test 17 round set: Average loss for 0.041 , Accuracy: 96%

Test 18 round set: Average loss for 0.037 , Accuracy: 96%

Test 19 round set: Average loss for 0.035 , Accuracy: 96%
```

```
Test 20 round set: Average loss for 0.034 , Accuracy: 97%
```

In [13]:
```
def NO_NMF_train2():
    # 1
    NO_NMF_model.train()
    for epoch in range(200):
        for batch_idx, (data, target) in enumerate(train_data2):
            optimizer.zero_grad()
            out = NO_NMF_model(data)
            loss = F.mse_loss(out[data.train_mask].flatten(), data.y[data
            loss.backward()
            optimizer.step()

def NO_NMF_test2():
    NO_NMF_model.eval()#2
    Pred,Real,Bias=[],[],[]
    with torch.no_grad():
        for data, target in test_data2:
            output = NO_NMF_model(data,A_tensor, X_tensor)
            test_loss += criterion(output, target).item()
            pred = output.argmax(1, keepdim=True)
            correct += pred.eq(target.view_as(pred)).sum().item()# 3
            real_values = data.y[data.test_mask]
            Mean = target.mean()-pred.mean()
            bias = (preds[data.test_mask] - real_values).mean()
            Pred.append(pred)
            Real.append(real_values)
            Bias.append(bias)
        mse = mean_squared_error(real_values.cpu().detach().numpy(), pred
        mae = mean_absolute_error(real_values.cpu().detach().numpy(), pre
        bias = (preds[data.test_mask] - real_values).mean()
        accuracy = 100. * correct / len(test_loader.dataset)
    test_loss /= len(test_data2.dataset)
    return mse ,mae,Bias,accuracy,test_loss,Pred,Real,Mean

round=20
NO_NMF_Mse2=[]
NO_NMF_Mae2=[]
NO_NMF_Bias2=[]
NO_NMF_Accuracy2=[]
NO_NMF_Test_loss2=[]
NO_NMF_Pred2=[]
NO_NMF_Real2=[]
NO_NMF_Mean2=[]
for i in range(round):
    NO_NMF_train2()
    NO_NMF_mse2,NO_NMF_mae2,NO_NMF_bias2,NO_NMF_accuracy2,NO_NMF_test_los
    NO_NMF_Mse2.appendNO_NMF_mse2)
    NO_NMF_Mae2.append(NO_NMF_mae2)
    NO_NMF_Bias2.append(NO_NMF_bias2)
    NO_NMF_Accuracy2.append(NO_NMF_accuracy2)
    NO_NMF_Test_loss2.append(NO_NMF_test_loss2)
    NO_NMF_Pred2.append(NO_NMF_pred2)
    NO_NMF_Real2.append(NO_NMF_real2)
    NO_NMF_Mean2.append(NO_NMF_mean2)
    print('\nTest {} round set: Average loss for {:.3f} , Accuracy: {:.0f
        i+1,NO_NMF_Test_loss2[i], NO_NMF_accuracy2[i]))
```

```
Test 1 round set: Average loss for 0.481 , Accuracy: 52%

Test 2 round set: Average loss for 0.422 , Accuracy: 60%

Test 3 round set: Average loss for 0.363 , Accuracy: 64%

Test 4 round set: Average loss for 0.336 , Accuracy: 67%

Test 5 round set: Average loss for 0.295 , Accuracy: 71%

Test 6 round set: Average loss for 0.238 , Accuracy: 77%

Test 7 round set: Average loss for 0.178 , Accuracy: 83%

Test 8 round set: Average loss for 0.155 , Accuracy: 85%

Test 9 round set: Average loss for 0.148 , Accuracy: 86%

Test 10 round set: Average loss for 0.132 , Accuracy: 87%

Test 11 round set: Average loss for 0.122 , Accuracy: 88%

Test 12 round set: Average loss for 0.116 , Accuracy: 89%

Test 13 round set: Average loss for 0.107 , Accuracy: 90%

Test 14 round set: Average loss for 0.095 , Accuracy: 90%

Test 15 round set: Average loss for 0.092 , Accuracy: 91%

Test 16 round set: Average loss for 0.085 , Accuracy: 92%

Test 17 round set: Average loss for 0.081 , Accuracy: 92%

Test 18 round set: Average loss for 0.077 , Accuracy: 92%

Test 19 round set: Average loss for 0.075 , Accuracy: 92%
```

```
Test 20 round set: Average loss for 0.074 , Accuracy: 93%
```

Our method without GCN

In [14]:
```
def nmf(R, Z, _lambda, T, learning_rate, decay_rate):
    num_users, num_items = R.shape
    num_features = Z.shape[1]
    P = np.random.rand(num_users, num_features)
    Q = np.random.rand(num_items, num_features)
    for t in range(T):
        for i in range(num_users):
            for j in range(num_items):
                if R[i, j] > 0:
                    e_ij = R[i, j] - np.dot(P[i, :], Q[j, :].T)
                    P[i, :] = P[i, :] + 2 * learning_rate * (e_ij * Q[j,
                    Q[j, :] = Q[j, :] + 2 * learning_rate * (e_ij * P[i,
        learning_rate = learning_rate * (1 - decay_rate)
    return P, Q

class NO_GCN(torch.nn.Module):
    def __init__(self, num_features, num_classes):
        super(NO_GCN, self).__init__()
        self.conv1 = GCNConv(num_features, 16)
        self.conv2 = GCNConv(16, num_classes)

    def forward(self, data):
        x, edge_index = data.x, data.edge_index
        x = self.conv1(x, edge_index)
        x = F.relu(x)
        x = self.conv2(x, edge_index)
        return x

R = np.array([[5, 3, 0, 1], [4, 0, 0, 1], [1, 1, 0, 5], [1, 0, 0, 4], [0,
Z = np.array([[0.1, 0.2], [0.2, 0.1], [0.3, 0.4], [0.4, 0.3], [0.5, 0.6]]
_lambda = 0.1
T = 100
learning_rate = 0.01
decay_rate = 0.01

P, Q = nmf(R, Z, _lambda, T, learning_rate, decay_rate)
num_features = P.shape[1] + Q.shape[1]
num_features = 64
num_classes = 1
NO_GCN_model = NO_GCN(num_features, num_classes)

optimizer = optim.Adam(NO_GCN_model.parameters(), lr=0.01)


A = np.zeros((R.shape[0], R.shape[1]))
for i in range(R.shape[0]):
    for j in range(R.shape[1]):
        if R[i, j] > 0:
            A[i, j] = 1
```

In [15]:
```
def NO_GCN_train1():
    NO_GCN_model.train()
    for epoch in range(200):
        for batch_idx, (data, target) in enumerate(train_data1):
            optimizer.zero_grad()
            input_data = torch.tensor(np.concatenate([P, Q], axis=1), dty
            target_data = torch.tensor(R, dtype=torch.float)
            out =NO_GCN_model(torch.tensor(A, dtype=torch.float), data)
            loss = F.mse_loss(out[data.train_mask].flatten(), data.y[data
            loss.backward()
            optimizer.step()

def NO_GCN_test1():
    NO_GCN_model.eval()#4
    Pred,Real,Bias=[],[],[]
    with torch.no_grad():
        for data, target in test_data1:
            output = NO_GCN_model(data)
            test_loss += criterion(output, target).item()
            pred = output.argmax(1, keepdim=True)
            correct += pred.eq(target.view_as(pred)).sum().item()# 2
            real_values = data.y[data.test_mask]
            Mean = target.mean()-pred.mean()
            bias = (preds[data.test_mask] - real_values).mean()
            Pred.append(pred)
            Real.append(real_values)
            Bias.append(bias)
        mse = mean_squared_error(real_values.cpu().detach().numpy(), pred
        mae = mean_absolute_error(real_values.cpu().detach().numpy(), pre
        bias = (preds[data.test_mask] - real_values).mean()
        accuracy = 100. * correct / len(test_data1.dataset)
    test_loss /= len(test_loader.dataset)
    return mse ,mae,bias,accuracy,test_loss,Pred,Real,Mean

round=20
NO_GCN_Mse1=[]
NO_GCN_Mae1=[]
NO_GCN_Bias1=[]
NO_GCN_Accuracy1=[]
NO_GCN_Test_loss1=[]
NO_GCN_Pred1=[]
NO_GCN_Real1=[]
NO_GCN_Mean1=[]
for i in range(round):
    NO_GCN_train1()
    NO_GCN_mse1,NO_GCN_mae1,NO_GCN_bias1,NO_GCN_accuracy1,NO_GCN_test_los
    NO_GCN_Mse1.appendNO_GCN_mse1)
    NO_GCN_Mae1.append(NO_GCN_mae1)
    NO_GCN_Bias1.append(NO_GCN_bias1)
    NO_GCN_Accuracy1.append(NO_GCN_accuracy1)
    NO_GCN_Test_loss1.append(NO_GCN_test_loss1)
    NO_GCN_Pred1.append(NO_GCN_pred1)
    NO_GCN_Real1.append(NO_GCN_real1)
    NO_GCN_Mean1.append(NO_GCN_mean1)
    print('\nTest {} round set: Average loss for {:.3f} , Accuracy: {:.0f
        i+1,NO_GCN_Test_loss1[i], NO_GCN_accuracy1[i]))
```

```
Test 1 round set: Average loss for 0.383 , Accuracy: 62%

Test 2 round set: Average loss for 0.291 , Accuracy: 71%

Test 3 round set: Average loss for 0.276 , Accuracy: 73%

Test 4 round set: Average loss for 0.245 , Accuracy: 76%

Test 5 round set: Average loss for 0.222 , Accuracy: 78%

Test 6 round set: Average loss for 0.218 , Accuracy: 80%

Test 7 round set: Average loss for 0.184 , Accuracy: 82%

Test 8 round set: Average loss for 0.171 , Accuracy: 83%

Test 9 round set: Average loss for 0.168 , Accuracy: 84%

Test 10 round set: Average loss for 0.162 , Accuracy: 84%

Test 11 round set: Average loss for 0.160 , Accuracy: 84%

Test 12 round set: Average loss for 0.159 , Accuracy: 85%

Test 13 round set: Average loss for 0.155 , Accuracy: 85%

Test 14 round set: Average loss for 0.145 , Accuracy: 86%

Test 15 round set: Average loss for 0.143 , Accuracy: 86%

Test 16 round set: Average loss for 0.137 , Accuracy: 86%

Test 17 round set: Average loss for 0.135 , Accuracy: 86%

Test 18 round set: Average loss for 0.134 , Accuracy: 87%

Test 19 round set: Average loss for 0.132 , Accuracy: 87%
```

```
Test 20 round set: Average loss for 0.131 , Accuracy: 87%
```

In [16]:

```
def NO_GCN_train2():
    NO_GCN_model.train()
    for epoch in range(200):
        for batch_idx, (data, target) in enumerate(train_data2):
            optimizer.zero_grad()
            input_data = torch.tensor(np.concatenate([P, Q], axis=1), dty
            target_data = torch.tensor(R, dtype=torch.float)
            out =NO_GCN_model(torch.tensor(A, dtype=torch.float), data)
            loss = F.mse_loss(out[data.train_mask].flatten(), data.y[data
            loss.backward()
            optimizer.step()

def NO_GCN_test2():
    NO_GCN_model.eval()#
    Pred,Real=[],[]
    with torch.no_grad():
        for data, target in test_data2:
            output = NO_GCN_model(data)
            test_loss += criterion(output, target).item()
            pred = output.argmax(1, keepdim=True)
            correct += pred.eq(target.view_as(pred)).sum().item()#
            real_values = data.y[data.test_mask]
            Mean = target.mean()-pred.mean()
            bias = (preds[data.test_mask] - real_values).mean()
            Pred.append(pred)
            Real.append(real_values)
            Bias.append(bias)
        mse = mean_squared_error(real_values.cpu().detach().numpy(), pred
        mae = mean_absolute_error(real_values.cpu().detach().numpy(), pre
        bias = (preds[data.test_mask] - real_values).mean()
        accuracy = 100. * correct / len(test_loader.dataset)
    test_loss /= len(test_data2.dataset)
    return mse ,mae,bias,accuracy,test_loss,Pred,Real,Mean

round=20
NO_GCN_Mse2=[]
NO_GCN_Mae2=[]
NO_GCN_Bias2=[]
NO_GCN_Accuracy2=[]
NO_GCN_Test_loss2=[]
NO_GCN_Pred2=[]
NO_GCN_Real2=[]
for i in range(round):
    NO_GCN_train2()
    NO_GCN_mse2,NO_GCN_mae2,NO_GCN_bias2,NO_GCN_accuracy2,NO_GCN_test_los
    NO_GCN_Mse2.appendNO_GCN_mse2)
    NO_GCN_Mae2.append(NO_GCN_mae2)
    NO_GCN_Bias2.append(NO_GCN_bias2)
    NO_GCN_Accuracy2.append(NO_GCN_accuracy2)
    NO_GCN_Test_loss2.append(NO_GCN_test_loss2)
    NO_GCN_Pred2.append(NO_GCN_pred2)
    NO_GCN_Real2.append(NO_GCN_real2)
    NO_GCN_Mean2.append(NO_GCN_mean2)
    print('\nTest {} round set: Average loss for {:.3f} , Accuracy: {:.0f
        i+1,NO_GCN_Test_loss2[i], NO_GCN_accuracy2[i]))
```

```
Test 1 round set: Average loss for 0.481 , Accuracy: 52%

Test 2 round set: Average loss for 0.413 , Accuracy: 59%

Test 3 round set: Average loss for 0.380 , Accuracy: 62%

Test 4 round set: Average loss for 0.353 , Accuracy: 65%

Test 5 round set: Average loss for 0.317 , Accuracy: 69%

Test 6 round set: Average loss for 0.265 , Accuracy: 74%

Test 7 round set: Average loss for 0.237 , Accuracy: 77%

Test 8 round set: Average loss for 0.223 , Accuracy: 78%

Test 9 round set: Average loss for 0.206 , Accuracy: 80%

Test 10 round set: Average loss for 0.195 , Accuracy: 81%

Test 11 round set: Average loss for 0.183 , Accuracy: 82%

Test 12 round set: Average loss for 0.175 , Accuracy: 82%

Test 13 round set: Average loss for 0.171 , Accuracy: 83%

Test 14 round set: Average loss for 0.165 , Accuracy: 84%

Test 15 round set: Average loss for 0.161 , Accuracy: 84%

Test 16 round set: Average loss for 0.157 , Accuracy: 84%

Test 17 round set: Average loss for 0.155 , Accuracy: 84%

Test 18 round set: Average loss for 0.154 , Accuracy: 85%

Test 19 round set: Average loss for 0.152 , Accuracy: 85%
```

```
Test 20 round set: Average loss for 0.151 , Accuracy: 85%
```

Traditional statistical methods

In [17]:

```
class Net(torch.nn.Module):
    def __init__(self, num_features, num_classes):
        super(Net, self).__init__()
        self.conv1 = GCNConv(num_features, 16)
        self.conv2 = GCNConv(16, num_classes)

    def forward(self, data):
        x, edge_index = data.x, data.edge_index

        x = self.conv1(x, edge_index)
        x = F.relu(x)
        x = self.conv2(x, edge_index)

        return x

device = torch.device('cuda' if torch.cuda.is_available() else 'cpu')
model = Net(dataset.num_node_features, 1).to(device)
optimizer = torch.optim.Adam(model.parameters(), lr=0.01, weight_decay=5e
```

In [18]:

```
def train1():
    # 1
    model.train()
    for epoch in range(200):
        for batch_idx, (data, target) in enumerate(train_data1):
            optimizer.zero_grad()
            out = model(data)
            loss = F.mse_loss(out[data.train_mask].flatten(), data.y[data
            loss.backward()
            optimizer.step()

def test1():
    model.eval()#测试模型
    Pred,Real,Bias=[],[],[]
    with torch.no_grad():
        for data, target in test_data1:
            output = model(data)
            test_loss += criterion(output, target).item()
            pred = output.argmax(1, keepdim=True)
            correct += pred.eq(target.view_as(pred)).sum().item()# 2
            real_values = data.y[data.test_mask]
            Mean = target.mean()-pred.mean()
            bias = (preds[data.test_mask] - real_values).mean()
            Pred.append(pred)
            Real.append(real_values)
            Bias.append(bias)
        mse = mean_squared_error(real_values.cpu().detach().numpy(), pred
        mae = mean_absolute_error(real_values.cpu().detach().numpy(), pre
        bias = (preds[data.test_mask] - real_values).mean()
        accuracy = 100. * correct / len(test_data1.dataset)
    test_loss /= len(test_loader.dataset)
```

```
    return mse ,mae,Bias,accuracy,test_loss,Pred,Real,Mean

round=20
Traditional_statistical_methods_Mse1=[]
Traditional_statistical_methods_Mae1=[]
Traditional_statistical_methods_Bias1=[]
Traditional_statistical_methods_Accuracy1=[]
Traditional_statistical_methods_Test_loss1=[]
Traditional_statistical_methods_Pred1=[]
Traditional_statistical_methods_Real1=[]
Traditional_statistical_methods_Mean1=[]
for i in range(round):
    train2()
    Traditional_statistical_methods_mse1,Traditional_statistical_methods_
    Traditional_statistical_methods_accuracy1,Traditional_statistical_met
    Traditional_statistical_methods_real1,Traditional_statistical_methods
    Traditional_statistical_methods_Mse1.append(Traditional_statistical_m
    Traditional_statistical_methods_Mae1.append(Traditional_statistical_m
    Traditional_statistical_methods_Bias1.append(Traditional_statistical_
    Traditional_statistical_methods_Accuracy1.append(Traditional_statisti
    Traditional_statistical_methods_Test_loss1.append(Traditional_statist
    Traditional_statistical_methods_Pred1.append(Traditional_statistical_
    Traditional_statistical_methods_Real1.append(Traditional_statistical_
    Traditional_statistical_methods_Mean1.append(Traditional_statistical_
    print('\nTest {} round set: Average loss for {:.3f} , Accuracy: {:.0f
        i+1,Traditional_statistical_methods_test_loss1[i], Traditional_st
```

```
Test 1 round set: Average loss for 0.384 , Accuracy: 62%


Test 2 round set: Average loss for 0.313 , Accuracy: 69%


Test 3 round set: Average loss for 0.305 , Accuracy: 70%


Test 4 round set: Average loss for 0.283 , Accuracy: 72%


Test 5 round set: Average loss for 0.255 , Accuracy: 75%


Test 6 round set: Average loss for 0.242 , Accuracy: 76%


Test 7 round set: Average loss for 0.241 , Accuracy: 76%


Test 8 round set: Average loss for 0.233 , Accuracy: 77%


Test 9 round set: Average loss for 0.225 , Accuracy: 78%


Test 10 round set: Average loss for 0.223 , Accuracy: 78%
```

```
Test 11 round set: Average loss for 0.217 , Accuracy: 78%

Test 12 round set: Average loss for 0.212 , Accuracy: 79%

Test 13 round set: Average loss for 0.207 , Accuracy: 79%

Test 14 round set: Average loss for 0.202 , Accuracy: 80%

Test 15 round set: Average loss for 0.199 , Accuracy: 80%

Test 16 round set: Average loss for 0.195 , Accuracy: 80%

Test 17 round set: Average loss for 0.192 , Accuracy: 81%

Test 18 round set: Average loss for 0.191 , Accuracy: 81%

Test 19 round set: Average loss for 0.187 , Accuracy: 81%

Test 20 round set: Average loss for 0.182 , Accuracy: 82%
```

In [19]:

```
def train2():
    # 2
    model.train()
    for epoch in range(200):
        for batch_idx, (data, target) in enumerate(train_data2):
            optimizer.zero_grad()
            out = model(data)
            loss = F.mse_loss(out[data.train_mask].flatten(), data.y[data
            loss.backward()
            optimizer.step()

def test2():
    model.eval()#4
    Pred,Real,Bias=[],[][]
    with torch.no_grad():
        for data, target in test_data2:
            output = model(data)
            test_loss += criterion(output, target).item()
            pred = output.argmax(1, keepdim=True)
            correct += pred.eq(target.view_as(pred)).sum().item()#
            real_values = data.y[data.test_mask]
            Mean = target.mean()-pred.mean()
            bias = (preds[data.test_mask] - real_values).mean()
            Pred.append(pred)
            Real.append(real_values)
            Bias.append(bias)
        mse = mean_squared_error(real_values.cpu().detach().numpy(), pred
```

```
        mae = mean_absolute_error(real_values.cpu().detach().numpy(), pre
        bias = (preds[data.test_mask] - real_values).mean()
        accuracy = 100. * correct / len(test_loader.dataset)
    test_loss /= len(test_data2.dataset)
    return mse ,mae,Bias,accuracy,test_loss,Pred,Real,Mean

round=20
Traditional_statistical_methods_Mse2=[]
Traditional_statistical_methods_Mae2=[]
Traditional_statistical_methods_Bias2=[]
Traditional_statistical_methods_Accuracy2=[]
Traditional_statistical_methods_Test_loss2=[]
Traditional_statistical_methods_Pred2=[]
Traditional_statistical_methods_Real2=[]
Traditional_statistical_methods_Mean2=[]
for i in range(round):
    train2()
    Traditional_statistical_methods_mse2,Traditional_statistical_methods_
    Traditional_statistical_methods_accuracy2,Traditional_statistical_met
    Traditional_statistical_methods_real2,raditional_statistical_methods_
    Traditional_statistical_methods_Mse2.append(Traditional_statistical_m
    Traditional_statistical_methods_Mae2.append(Traditional_statistical_m
    Traditional_statistical_methods_Bias2.append(Traditional_statistical_
    Traditional_statistical_methods_Accuracy2.append(Traditional_statisti
    Traditional_statistical_methods_Test_loss2.append(Traditional_statist
    Traditional_statistical_methods_Pred2.append(Traditional_statistical_
    Traditional_statistical_methods_Real2.append(Traditional_statistical_
    Traditional_statistical_methods_Mean2.append(Traditional_statistical_
    print('\nTest {} round set: Average loss for {:.3f} , Accuracy: {:.0f
        i+1,Traditional_statistical_methods_test_loss2[i], Traditional_st
```

```
Test 1 round set: Average loss for 0.481 , Accuracy: 52%


Test 2 round set: Average loss for 0.422 , Accuracy: 58%


Test 3 round set: Average loss for 0.393 , Accuracy: 61%


Test 4 round set: Average loss for 0.396 , Accuracy: 61%


Test 5 round set: Average loss for 0.366 , Accuracy: 64%


Test 6 round set: Average loss for 0.353 , Accuracy: 65%


Test 7 round set: Average loss for 0.348 , Accuracy: 66%


Test 8 round set: Average loss for 0.335 , Accuracy: 67%


Test 9 round set: Average loss for 0.325 , Accuracy: 70%
```

```
Test 10 round set: Average loss for 0.286 , Accuracy: 72%

Test 11 round set: Average loss for 0.277 , Accuracy: 73%

Test 12 round set: Average loss for 0.265 , Accuracy: 74%

Test 13 round set: Average loss for 0.261 , Accuracy: 74%

Test 14 round set: Average loss for 0.257 , Accuracy: 74%

Test 15 round set: Average loss for 0.250 , Accuracy: 74%

Test 16 round set: Average loss for 0.254 , Accuracy: 75%

Test 17 round set: Average loss for 0.252 , Accuracy: 75%

Test 18 round set: Average loss for 0.251 , Accuracy: 75%

Test 19 round set: Average loss for 0.247 , Accuracy: 75%

Test 20 round set: Average loss for 0.245 , Accuracy: 75%
```

In [20]:
```
num_vars = 10
np.random.seed(0)
angles = np.linspace(0, 2 * np.pi, num_vars, endpoint=False).tolist()
Oursmethod = np.concatenate((Our_Module_mse1, [Our_Module_mse1[0]]))
OursmethodwithoutNMF = np.concatenate((NO_NMF_mse1, [NO_NMF_mse1[0]]))
OursmethodwithoutNMFGCN = np.concatenate((NO_GCN_mse1, [NO_GCN_mse1[0]]))
Traditionalstatisticalmethods = np.concatenate((Traditional_statistical_m
angles += angles[:1]

fig, ax = plt.subplots(figsize=(10, 10), subplot_kw=dict(polar=True))
ax.plot(angles, Oursmethod, color='red', linewidth=2, label='Our method')
ax.fill(angles, Oursmethod, color='red', alpha=0.1)

ax.plot(angles, OursmethodwithoutNMF, color='deepskyblue', linewidth=2, l
ax.fill(angles, OursmethodwithoutNMF , color='deepskyblue', alpha=0.1)

ax.plot(angles, OursmethodwithoutNMFGCN, color='forestgreen', linewidth=2
ax.fill(angles, OursmethodwithoutNMFGCN, color='forestgreen', alpha=0.1)

ax.plot(angles, Traditionalstatisticalmethods, color='darkorange', linewi
ax.fill(angles, Traditionalstatisticalmethods, color='darkorange', alpha=

ax.set_theta_offset(np.pi / 2)
ax.set_theta_direction(-1)
labels = ['Metric 1', 'Metric 2', 'Metric 3', 'Metric 4', 'Metric 5', 'Me
ax.set_thetagrids(np.degrees(angles), labels, fontsize=12)
ax.legend(loc='upper right', bbox_to_anchor=(1.1, 1.1), fontsize=12)
sns.set(style='whitegrid',font_scale=1.5)
plt.savefig("3.pdf")
plt.show()
```

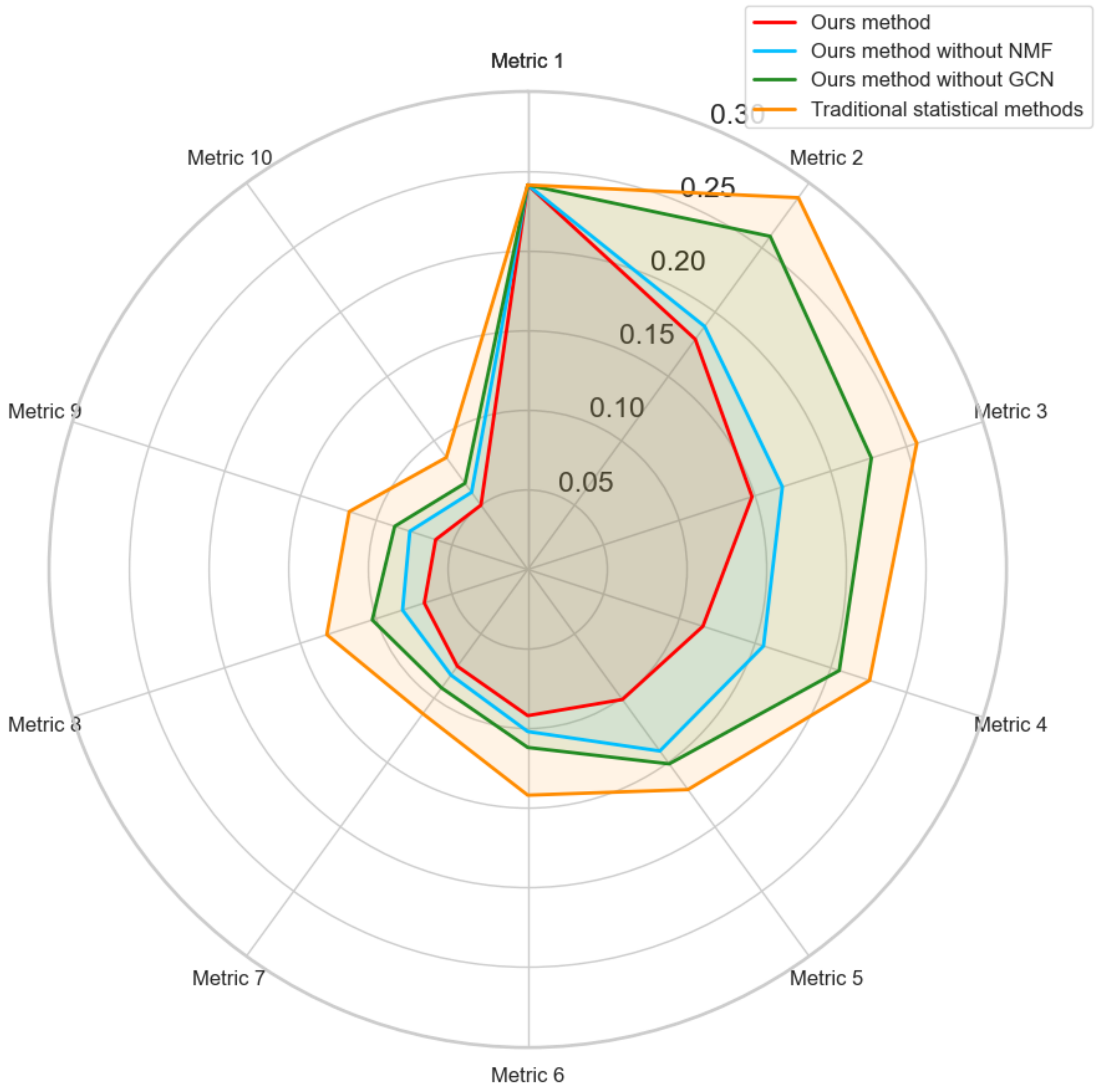
Ours method
Ours method without NMF
Ours method without GCN
Traditional statistical methods
Metric 1
Metric 2
Metric 3
Metric 4
Metric 5
Metric 6
Metric 7
Metric 8
Metric 9
Metric 10
0.05
0.10
0.15
0.20
0.25

In [21]:
```
num_vars = 10
np.random.seed(0)
angles = np.linspace(0, 2 * np.pi, num_vars, endpoint=False).tolist()
Oursmethod = np.concatenate((Our_Module_mse2, [Our_Module_mse2[0]]))
OursmethodwithoutNMF = np.concatenate((NO_NMF_mse2, [NO_NMF_mse2[0]]))
OursmethodwithoutNMFGCN = np.concatenate((NO_GCN_mse2, [NO_GCN_mse2[0]]))
Traditionalstatisticalmethods = np.concatenate((Traditional_statistical_m
angles += angles[:1]
fig, ax = plt.subplots(figsize=(10, 10), subplot_kw=dict(polar=True))
ax.plot(angles, Oursmethod, color='red', linewidth=2, label='Our method')
ax.fill(angles, Oursmethod, color='red', alpha=0.1)
ax.plot(angles, OursmethodwithoutNMF, color='deepskyblue', linewidth=2, l
ax.fill(angles, OursmethodwithoutNMF , color='deepskyblue', alpha=0.1)
ax.plot(angles, OursmethodwithoutNMFGCN, color='forestgreen', linewidth=2
ax.fill(angles, OursmethodwithoutNMFGCN, color='forestgreen', alpha=0.1)
ax.plot(angles, Traditionalstatisticalmethods, color='darkorange', linewi
ax.fill(angles, Traditionalstatisticalmethods, color='darkorange', alpha=
ax.set_theta_offset(np.pi / 2)
ax.set_theta_direction(-1)
labels = ['Metric 1', 'Metric 2', 'Metric 3', 'Metric 4', 'Metric 5', 'Me
ax.set_thetagrids(np.degrees(angles), labels, fontsize=12)
ax.legend(loc='upper right', bbox_to_anchor=(1.1, 1.1), fontsize=12)
sns.set(style='whitegrid',font_scale=1.5)
plt.savefig("3.pdf")
plt.show()
```

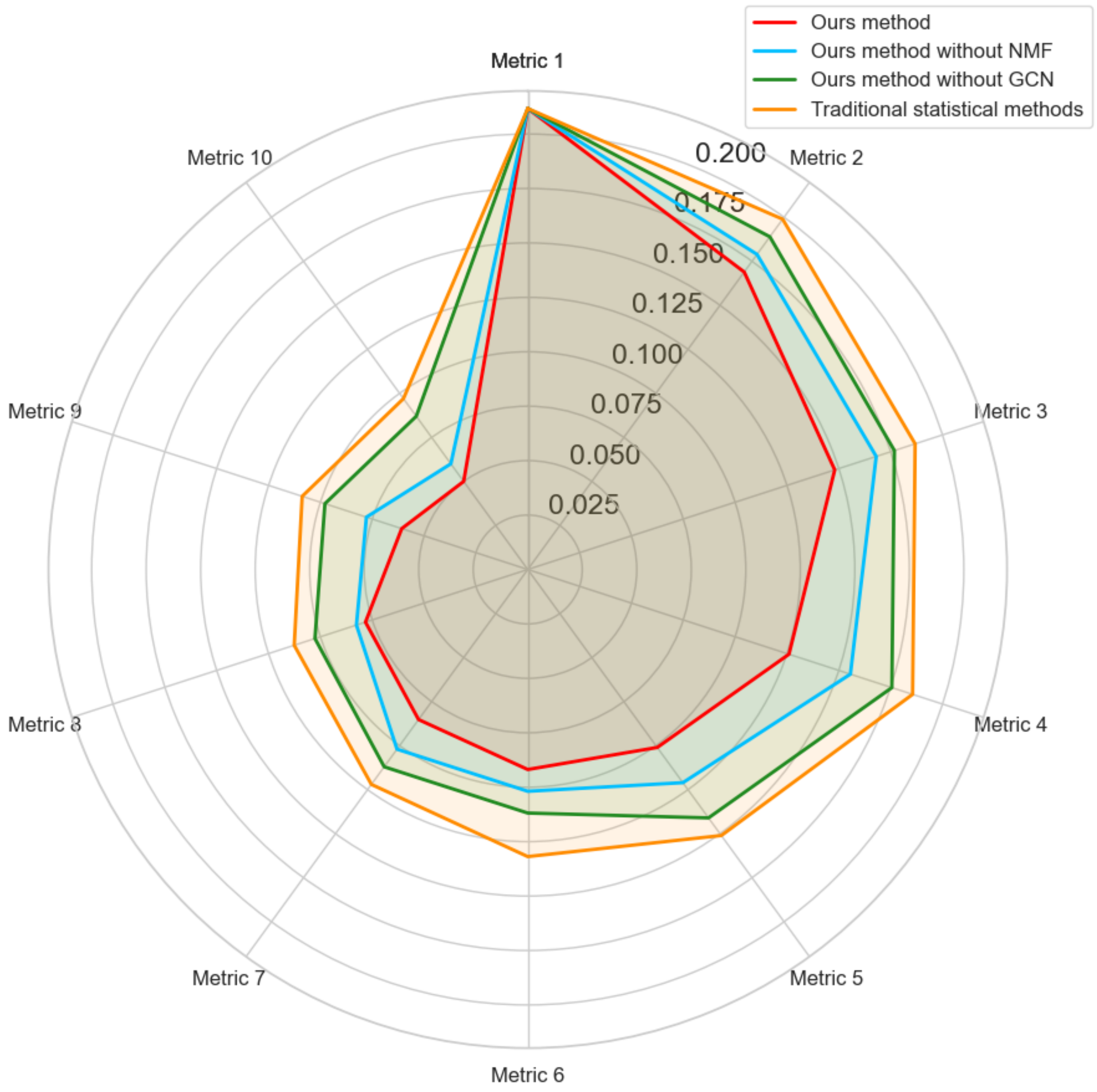

In [22]:

```
data = np.array([Our_Module_mae1, NO_NMF_mae1, NO_GCN_mae1,Traditional_st
fig, ax = plt.subplots(figsize=(10, 6))
sns.heatmap(data, annot=True, fmt="d", linewidths=.5, ax=ax, cmap='YlGnBu
ax.set_title('Comparison of Different Methods', fontsize=16)
plt.savefig("5.pdf", bbox_inches='tight')
plt.show()
```

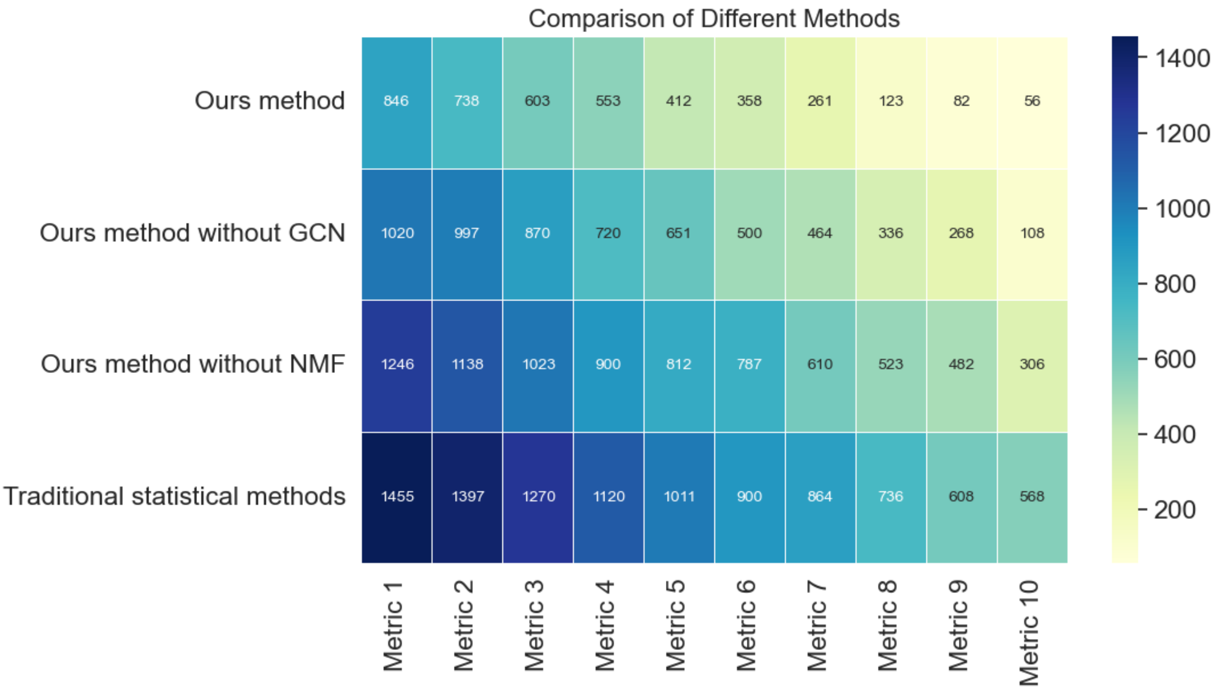

In [23]:

```
data = np.array([Our_Module_mae2, NO_NMF_mae2, NO_GCN_mae2,Traditional_st
fig, ax = plt.subplots(figsize=(10, 6))
sns.heatmap(data, annot=True, fmt="d", linewidths=.5, ax=ax, cmap='YlGnBu
ax.set_title('Comparison of Different Methods', fontsize=16)
plt.savefig("6.pdf", bbox_inches='tight')
plt.show()
```

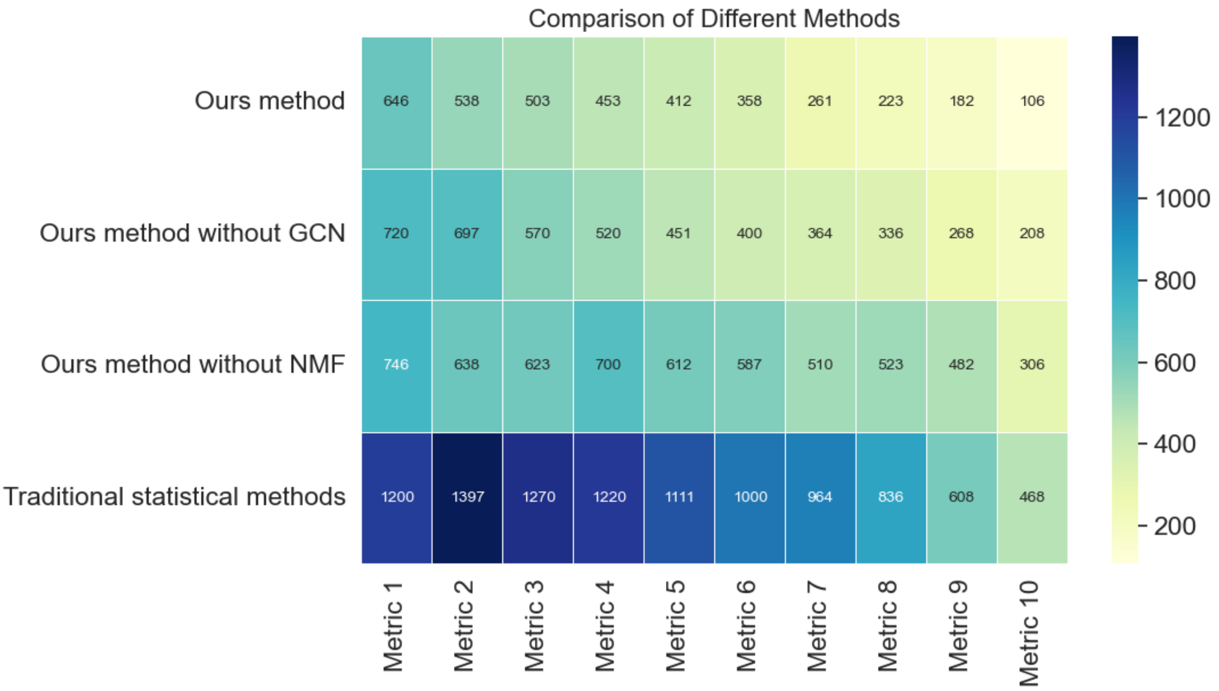

In [24]:
```
df = pd.DataFrame({
    'Our method': Our_Module_Bias1,
    'Our method without NMF': NO_NMF_Bias1,
    'Our method without GCN': NO_GCN_Bias1,
    'Traditional statistical methods': Traditional_statistical_methods_Bi
})

plt.figure(figsize=(10,6))
for column in df.columns:
    sns.kdeplot(df[column], shade=True, label=column)
plt.xlabel('Values')
plt.ylabel('Density')
plt.title('Comparison of Different Methods')
plt.legend(fontsize='small')
plt.savefig("7.pdf")
plt.show()
```

```
C:\Users\17401\anaconda3\envs\torch\lib\site-packages\ipykernel_launcher.
py:24: FutureWarning:

`shade` is now deprecated in favor of `fill`; setting `fill=True`.
This will become an error in seaborn v0.14.0; please update your code.

C:\Users\17401\anaconda3\envs\torch\lib\site-packages\ipykernel_launcher.
py:24: FutureWarning:

`shade` is now deprecated in favor of `fill`; setting `fill=True`.
This will become an error in seaborn v0.14.0; please update your code.

C:\Users\17401\anaconda3\envs\torch\lib\site-packages\ipykernel_launcher.
py:24: FutureWarning:

`shade` is now deprecated in favor of `fill`; setting `fill=True`.
This will become an error in seaborn v0.14.0; please update your code.

C:\Users\17401\anaconda3\envs\torch\lib\site-packages\ipykernel_launcher.
py:24: FutureWarning:

`shade` is now deprecated in favor of `fill`; setting `fill=True`.
This will become an error in seaborn v0.14.0; please update your code.
```

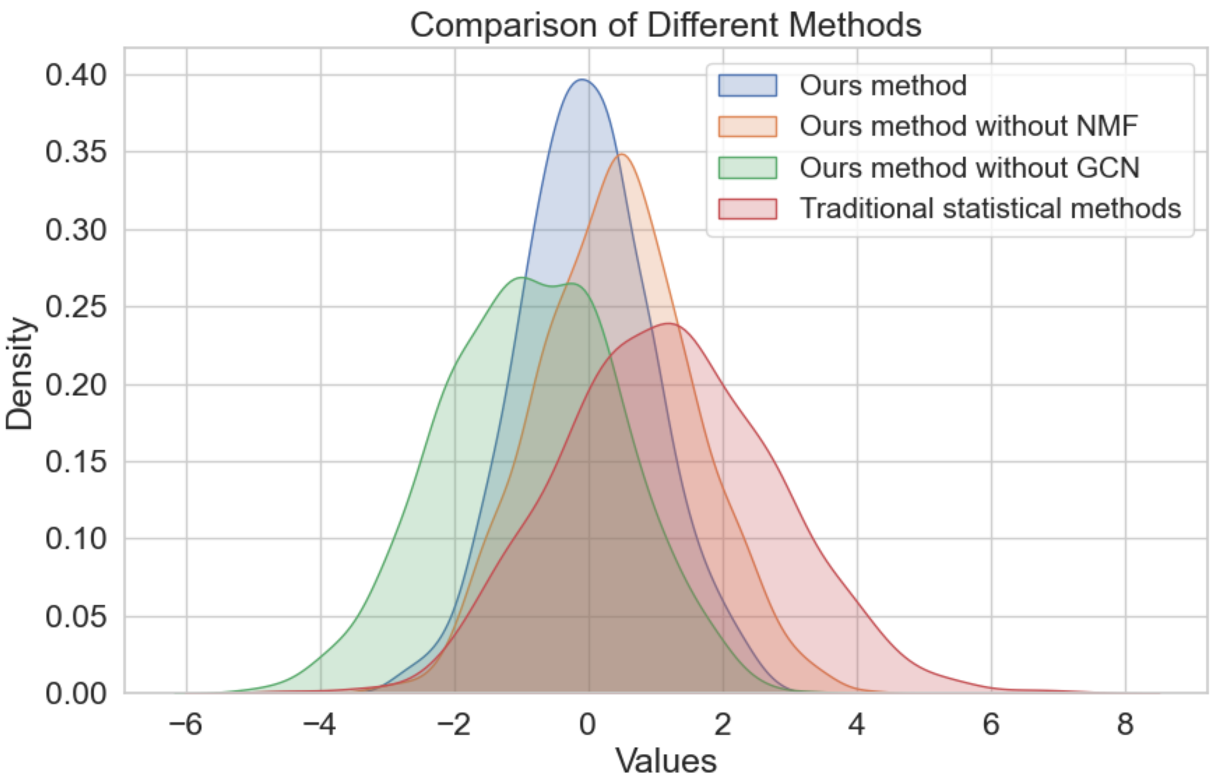

In [25]:
```
df = pd.DataFrame({
    'Our method': Our_Module_Bias2,
    'Our method without NMF': NO_NMF_Bias2,
    'Our method without GCN': NO_GCN_Bias2,
    'Traditional statistical methods': Traditional_statistical_methods_Bi
})

# 创建密度图
plt.figure(figsize=(10,6))
for column in df.columns:
    sns.kdeplot(df[column], shade=True, label=column)

plt.xlabel('Values')
plt.ylabel('Density')
plt.title('Comparison of Different Methods')
plt.legend(fontsize='x-small', loc='upper left')

plt.savefig("8.pdf")
plt.show()
```

```
C:\Users\17401\anaconda3\envs\torch\lib\site-packages\ipykernel_launcher.
py:24: FutureWarning:

`shade` is now deprecated in favor of `fill`; setting `fill=True`.
This will become an error in seaborn v0.14.0; please update your code.

C:\Users\17401\anaconda3\envs\torch\lib\site-packages\ipykernel_launcher.
py:24: FutureWarning:

`shade` is now deprecated in favor of `fill`; setting `fill=True`.
This will become an error in seaborn v0.14.0; please update your code.

C:\Users\17401\anaconda3\envs\torch\lib\site-packages\ipykernel_launcher.
py:24: FutureWarning:

`shade` is now deprecated in favor of `fill`; setting `fill=True`.
This will become an error in seaborn v0.14.0; please update your code.

C:\Users\17401\anaconda3\envs\torch\lib\site-packages\ipykernel_launcher.
py:24: FutureWarning:

`shade` is now deprecated in favor of `fill`; setting `fill=True`.
This will become an error in seaborn v0.14.0; please update your code.
```

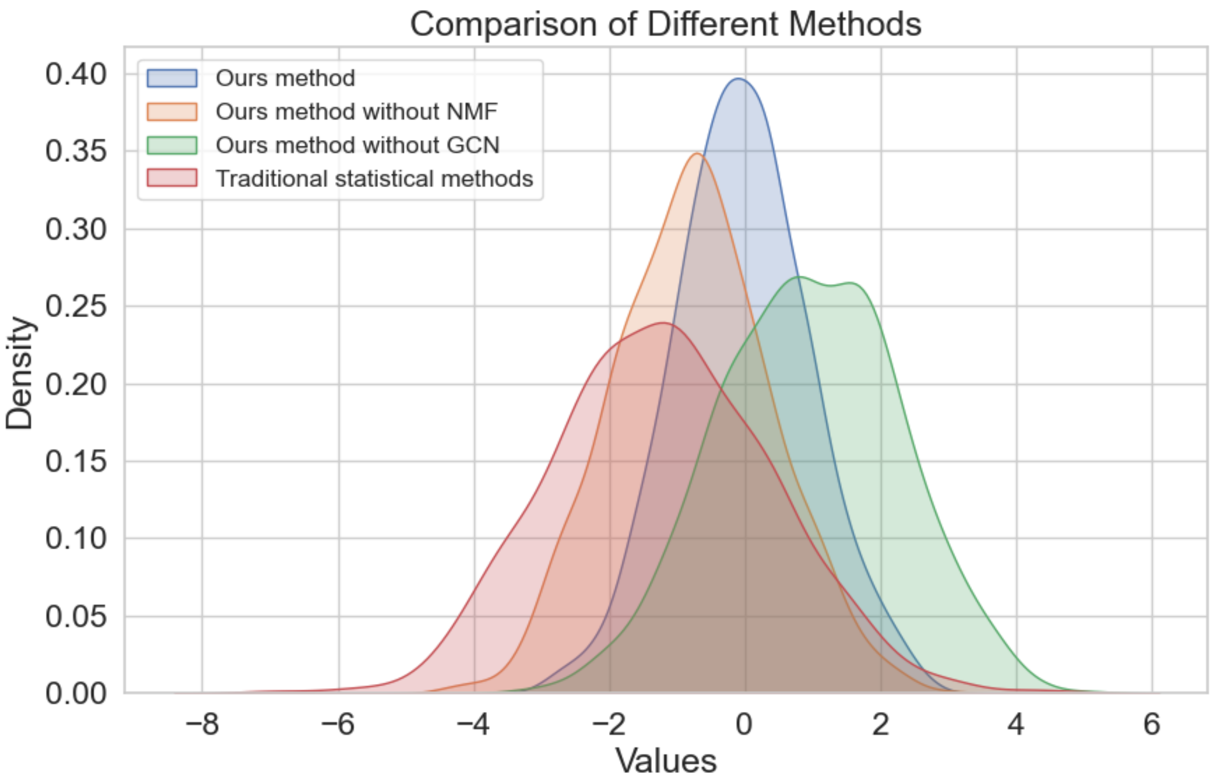

In [26]:
```
dataset1 = {
    'True value': Our_Module_Real,
    'Our method': Our_Module_Pred1,
    'Our method without NMF': NO_NMF_Pred1,
    'Our method without GCN': NO_GCN_Pred1,
    'Traditional statistical methods': Traditional_statistical_methods_Pr
}
df = pd.DataFrame(dataset1)
colors = ["darkorange", "red", "deepskyblue", "forestgreen", "gray"]
plt.figure(figsize=(12, 6))
sns.boxplot(data=df,palette=colors)
plt.xlabel('Methods')
plt.ylabel('Values')
sns.set(style='whitegrid',font_scale=1.5)
plt.tight_layout()
plt.savefig("9.pdf")
plt.show()
```

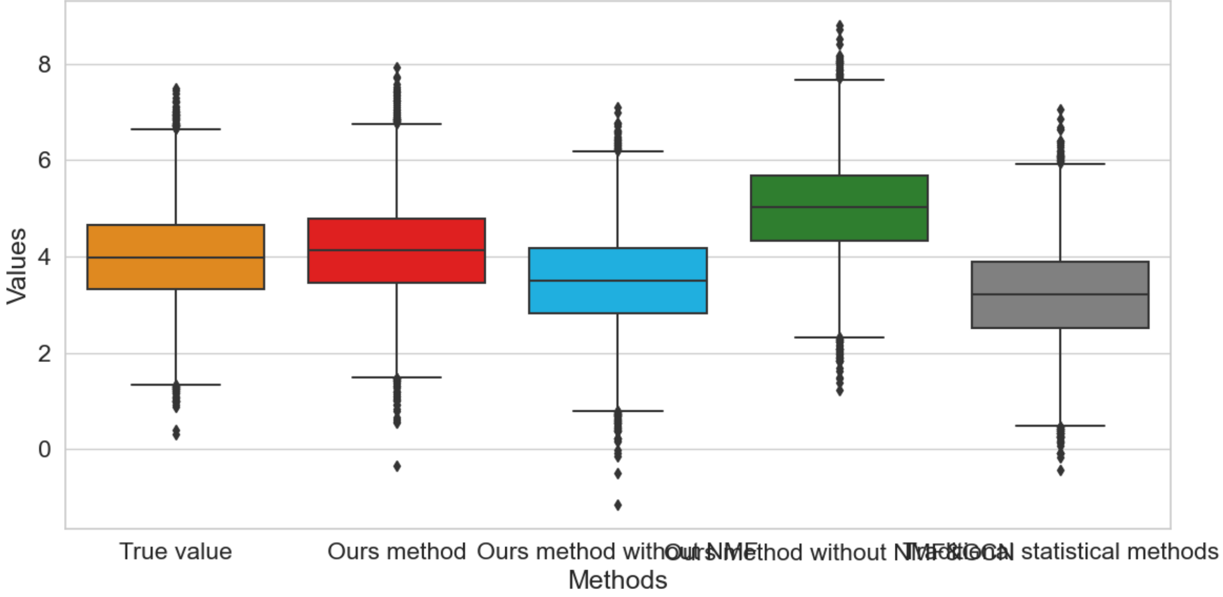

In [27]:
```
dataset2 = {
    'True value': Our_Module_Rea2,
    'Our method': Our_Module_Pred2,
    'Our method without NMF': NO_NMF_Pred2,
    'Our method without GCN': NO_GCN_Pred2,
    'Traditional statistical methods': Traditional_statistical_methods_Pr
}
df2 = pd.DataFrame(dataset2)
plt.figure(figsize=(12, 6))
colors = ["darkorange", "red", "deepskyblue", "forestgreen", "gray"]
sns.boxplot(data=df2, palette=colors)
plt.xlabel('Methods')
plt.ylabel('Values')
sns.set(style='whitegrid',font_scale=1.5)
plt.tight_layout()
plt.savefig("10.pdf")
plt.show()
```

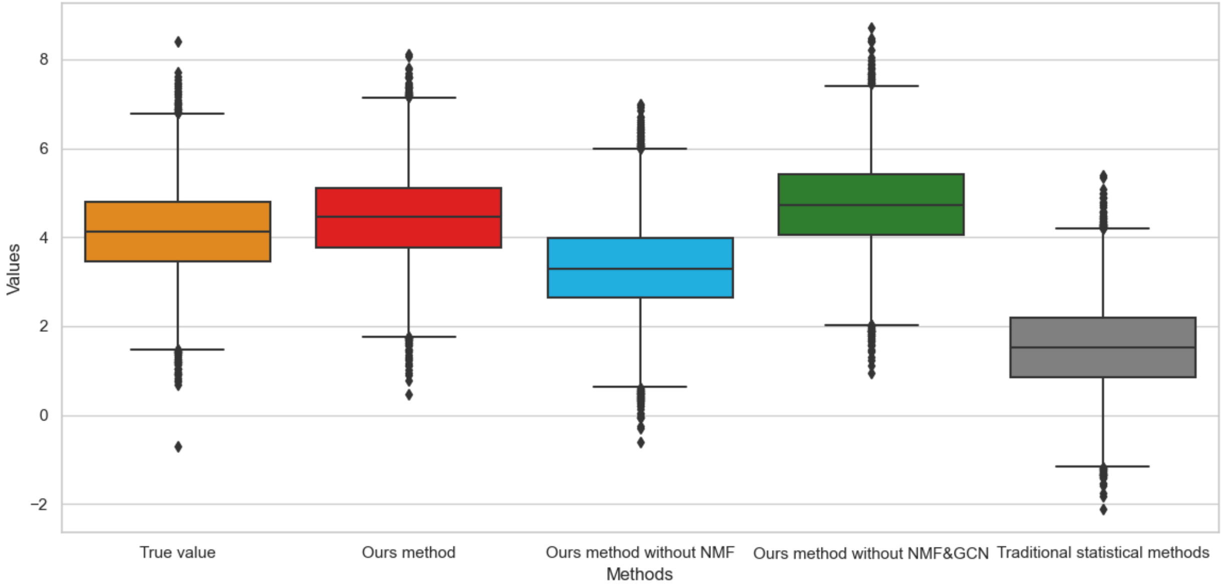

In [28]:

```
a = np.arange(1, len(Our_Module_Mean1) + 1)
width = 0.15
fig, ax = plt.subplots(figsize=(10, 6))
colors = ['blue', 'orange', 'purple', 'gray']
patterns = ['/', 'O', '.', '']
rects1 = ax.bar(a - (width * 2), Our_Module_Mean1, width, color=colors[0]
rects2 = ax.bar(a - width, NO_NMF_Mean1, width, color=colors[1], label='O
rects3 = ax.bar(a, NO_GCN__Mean1, width, color=colors[2], label='Our meth
rects4 = ax.bar(a + width, Traditional_statistical_methods_Mean1, width,
ax.set_xlabel('Bin ID')
ax.set_ylabel('Rating')
ax.set_xticks(a)
ax.set_xticklabels(a)
ax.legend(loc='upper left')
ax.grid(True)
plt.tight_layout()
plt.savefig("11.pdf")
plt.show()
```

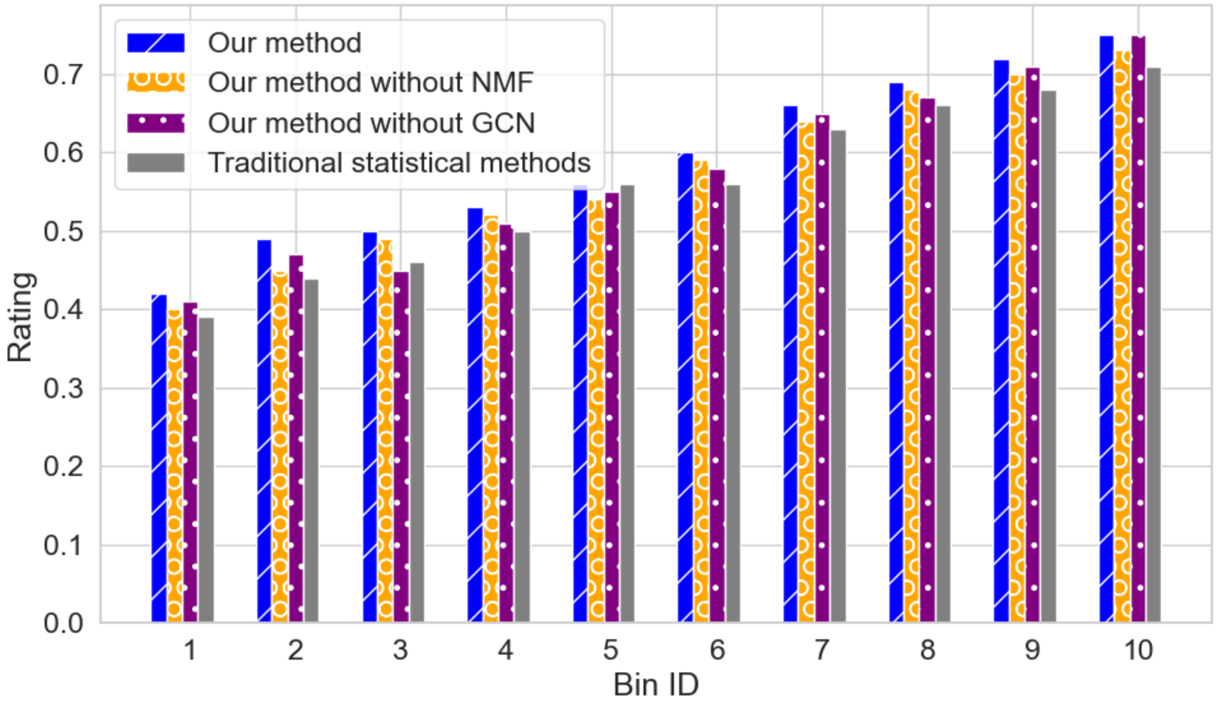

In [28]:

```
a = np.arange(1, len(Our_Module_Mean2) + 1)
width = 0.15
fig, ax = plt.subplots(figsize=(10, 6))
colors = ['blue', 'orange', 'purple', 'gray']
patterns = ['/', 'O', '.', '']
rects1 = ax.bar(a - (width * 2), Our_Module_Mean2, width, color=colors[0]
rects2 = ax.bar(a - width, NO_NMF_Mean2, width, color=colors[1], label='O
rects3 = ax.bar(a, NO_GCN__Mean2, width, color=colors[2], label='Our meth
rects4 = ax.bar(a + width, Traditional_statistical_methods_Mean2, width,
ax.set_xlabel('Bin ID')
ax.set_ylabel('Rating')
ax.set_xticks(a)
ax.set_xticklabels(a)
ax.legend(loc='upper left')
ax.grid(True)
plt.tight_layout()
plt.savefig("12.pdf")
plt.show()
```

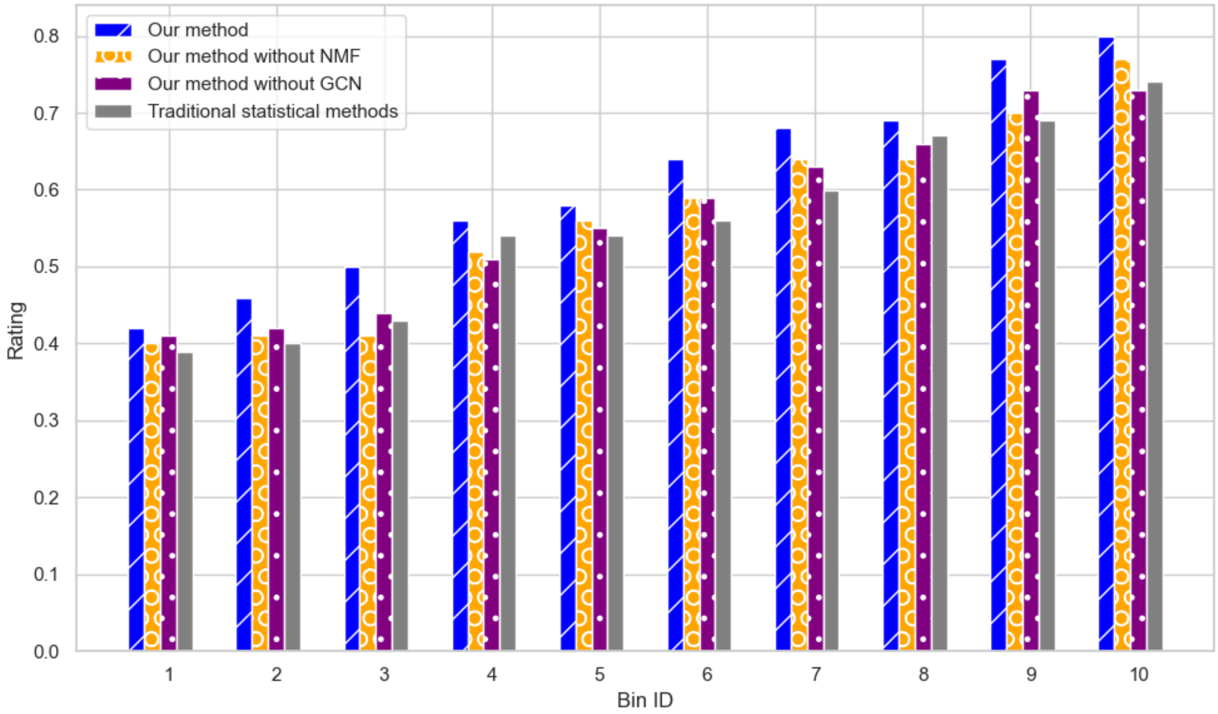

In [29]:

```
x = np.arange(1, len(Traditional_statistical_methods_accuracy1) + 1)
fig, ax = plt.subplots(figsize=(10, 6))
palette = sns.color_palette("muted")
ax.plot(x, Our_Module_accuracy1, marker='*', linewidth=2, linestyle='--',
ax.plot(x, NO_NMF_accuracy1, marker='o', linewidth=2, linestyle='dashdot'
ax.plot(x, NO_GCN_accuracy1, marker='v', linewidth=2, linestyle='-', colo
ax.plot(x, Traditional_statistical_methods_accuracy1, marker='^', linewid
ax.set_xlabel('Rounds',fontsize=12)
ax.set_ylabel('Accuracy',fontsize=15)
ax.legend(loc='lower right', fontsize=10)
ax.grid(True)
ax.xaxis.set_major_locator(plt.MaxNLocator(integer=True))
plt.tight_layout()
plt.savefig("13.pdf")
plt.show()
```

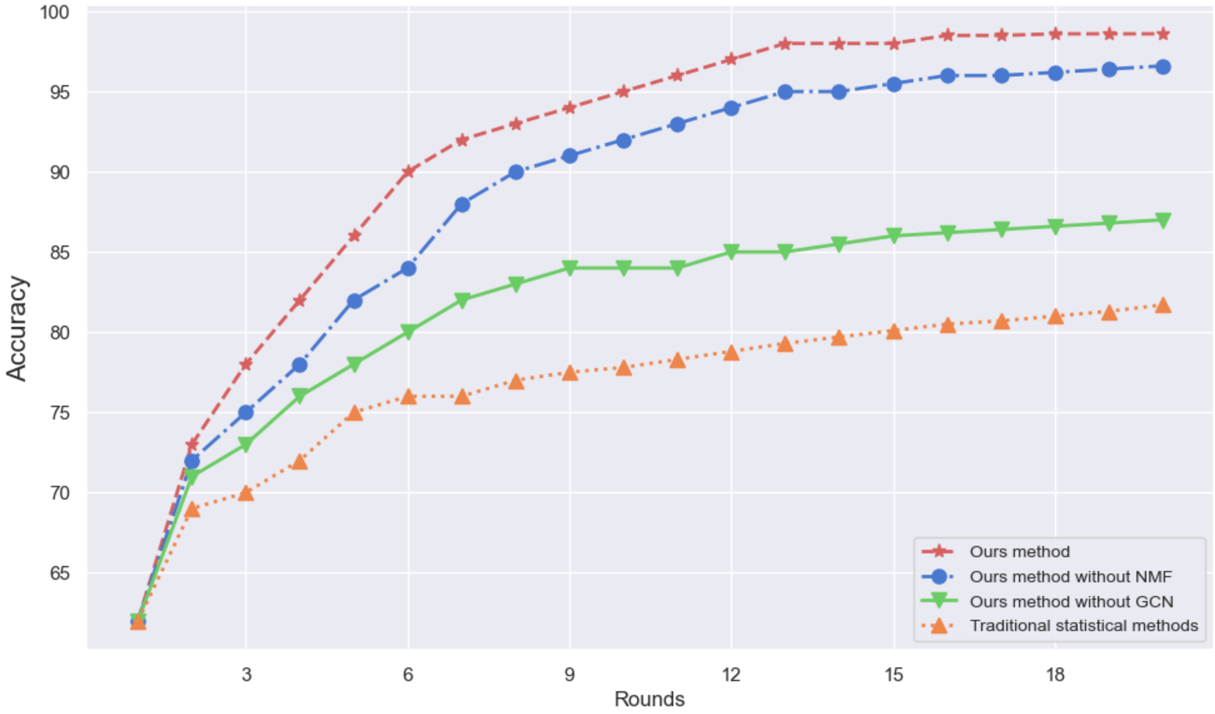

In [30]:
```
x = np.arange(1, len(Traditional_statistical_methods_accuracy2) + 1)
fig, ax = plt.subplots(figsize=(10, 6))
palette = sns.color_palette("muted")
ax.plot(x, Our_Module_accuracy2, marker='*', linewidth=2, linestyle='--',
ax.plot(x, NO_NMF_accuracy2, marker='o', linewidth=2, linestyle='dashdot'
ax.plot(x, NO_GCN_accuracy2, marker='v', linewidth=2, linestyle='-', colo
ax.plot(x, Traditional_statistical_methods_accuracy2, marker='^', linewid
ax.set_xlabel('Rounds',fontsize=15)
ax.set_ylabel('Accuracy',fontsize=15)
ax.legend(loc='lower right', fontsize=10)
ax.grid(True)
ax.xaxis.set_major_locator(plt.MaxNLocator(integer=True))
plt.tight_layout()
plt.savefig("14.pdf")
plt.show()
```

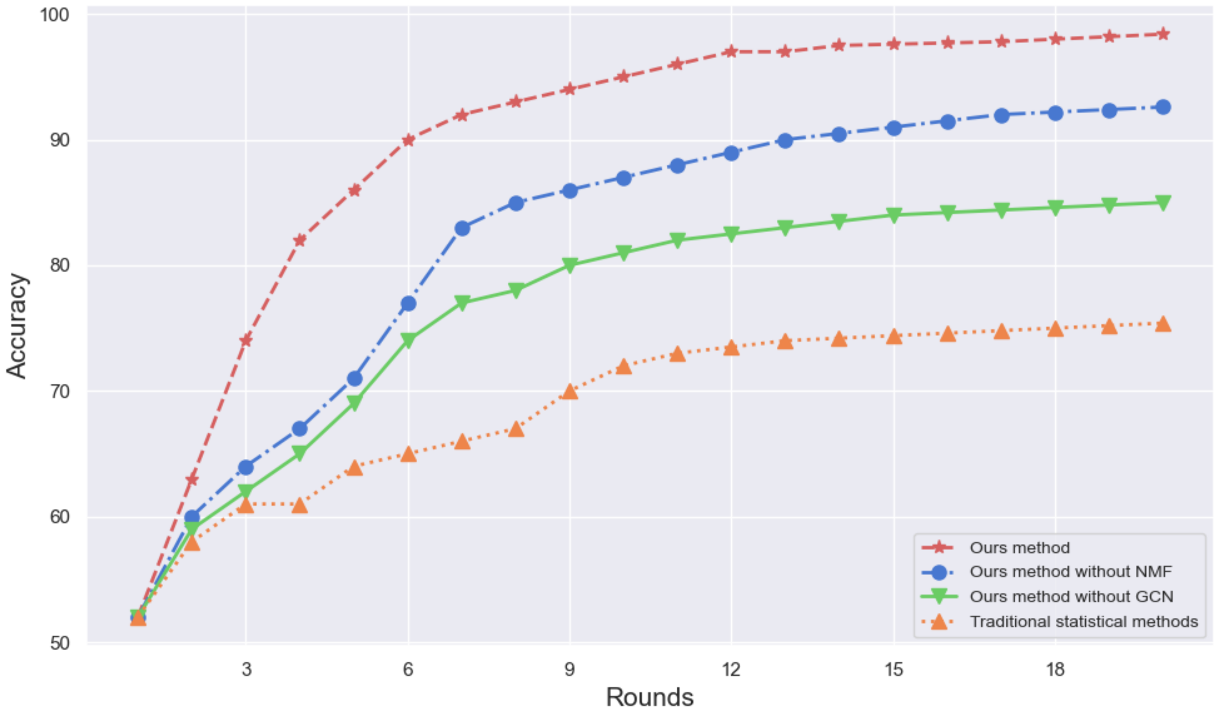
100
90
80
70
60
50
Accuracy
3
6
9
12
15
18
Rounds
Ours method
Ours method without NMF
Ours method without GCN
Traditional statistical methods
